# Thermal Relaxation in Janus Transition Metal Dichalcogenide Bilayers

**DOI:** 10.3390/ma17174200

**Published:** 2024-08-25

**Authors:** Aristotelis P. Sgouros, Fotios I. Michos, Michail M. Sigalas, George Kalosakas

**Affiliations:** 1 School of Chemical Engineering, National Technical University of Athens (NTUA), GR-15780 Athens, Greece; 2 Theoretical and Physical Chemistry Institute, National Hellenic Research Foundation, Vass. Constantinou 48, GR-11635 Athens, Greece; 3 Department of Materials Science, University of Patras, GR-26504 Patras, Greece; fotismixos@gmail.com (F.I.M.); sigalas@upatras.gr (M.M.S.)

**Keywords:** thermal transport, thermal conductivity, heterostructures, phonons, two-dimensional materials, out-of-equilibrium dynamics

## Abstract

In this work, we employ molecular dynamics simulations with semi-empirical interatomic potentials to explore heat dissipation in Janus transition metal dichalcogenides (JTMDs). The middle atomic layer is composed of either molybdenum (Mo) or tungsten (W) atoms, and the top and bottom atomic layers consist of sulfur (S) and selenium (Se) atoms, respectively. Various nanomaterials have been investigated, including both pristine JTMDs and nanostructures incorporating inner triangular regions with a composition distinct from the outer bulk material. At the beginning of our simulations, a temperature gradient across the system is imposed by heating the central region to a high temperature while the surrounding area remains at room temperature. Once a steady state is reached, characterized by a constant energy flux, the temperature control in the central region is switched off. The heat attenuation is investigated by monitoring the characteristic relaxation time (*τ*_av_) of the local temperature at the central region toward thermal equilibrium. We find that SMoSe JTMDs exhibit thermal attenuation similar to conventional TMDs (*τ*_av_~10–15 ps). On the contrary, SWSe JTMDs feature relaxation times up to two times as high (*τ*_av_~14–28 ps). Forming triangular lateral heterostructures in their surfaces leads to a significant slowdown in heat attenuation by up to about an order of magnitude (*τ*_av_~100 ps).

## 1. Introduction

Two-dimensional (2D) materials with a few-atoms thickness have captured significant attention due to their unique properties arising from their planar structure and large relative surface area, giving rise to exceptional electronic, mechanical, thermal, optical, and chemical properties [1,2,3,4,5,6,7,8,9]. The electrical conductivity of 2D materials varies widely, encompassing insulating materials from hexagonal boron nitride (h-BN), to semiconducting transition metal dichalcogenides (TMDs) [10,11], to conducting transition metal carbides/nitrides (MXenes) [4,12].

The electronic properties of TMD materials and the corresponding band gaps (BGs) can be tuned by adjusting their thickness [13,14,15,16], by varying the stoichiometry of their alloys [17,18,19,20,21], by imposing strain [5,22,23], and through functionalization [24,25,26].

For example, monolayer TMDs with composition MX_2_, where M is a transition-metal atom and X is a chalcogen atom (i.e., such as MoS_2_, MoSe_2_, WS_2_, and WSe_2_), exhibit a direct BG ranging between 1.1 and 2.0 eV at the K point of the Brillouin zone [22,23,27]. Increasing the thickness of TMDs, on the other hand, has been shown to yield indirect band gaps [13,14,16]. The aforementioned properties make the family of atomic thickness TMDs useful in various applications such as fast photodetection, photonic applications (including optical limiting and optical switching applications), and nanoelectromechanical devices (NEMS) such as pressure sensors and more [28,29,30].

Moreover, the electronic properties of these materials can be tuned by adjusting their stoichiometric composition. For example, photoluminescence characterization in conjunction with density functional theory calculations by Chen et al. [17] demonstrate that tunable band gaps can be obtained in Mo_1−*x*_W*_x_*S_2_ monolayer alloys (where *x* ranges from 0 to 1) by alloying two materials with different BGs (WS_2_ and MoS_2_). The optical band gap of Mo_1−*x*_W*_x_*S_2_ has also been measured by Pelaez-Fernandez et al. [21] by means of non-penetrative low-loss EELS analyses, in agreement with Chen et al. [17]. Li et al. [18], on the other hand, reported the simultaneous growth of MoS_2*x*_Se_2(1−*x*)_ triangular nanosheets with precise composition tunability. High-quality in-plane heterojunctions can be formed between monolayer MoSe_2_ and WSe_2_ [19], while band gaps varying between 1.82 eV (680 nm) to 1.64 eV (755 nm) have been found in bilayer MoS_2*x*_Se_2(1−*x*)_ alloys along single triangular nanosheets [20].

The past decade has witnessed a surge in applications exploiting the unique properties and characteristics of TMDs, such as in biomedical devices [31,32,33,34,35], photovoltaic measurements with p-n junctions and the controlled implantation of charge donors and acceptors in silicon crystals to form p–n junctions [36], p-n heterojunctions with MoS_2_ in n-type semiconductors [37], high-performance light-emitting transistors and field-effect transistors based on MoS_2_ and WS_2_ [38,39], and efficient solar cells based on the excellent properties of MoS_2_ [40,41].

Recently, a new class of hybrid TMDs has been discovered, the Janus transition metal dichalcogenides (JTMDs), featuring unique structures with distinct chalcogen atoms on their top and bottom layers [42,43,44,45,46]. Janus particles [47] constitute a class of colloidal particles that exhibit multiple distinct physical properties on their surfaces. The same concept has been transferred recently to characterize 2D materials with different chemical constitution/functionalization on their opposing sides. JTMDs are considered Janus in the sense that they break the vertical mirror symmetry [42,43,44,45,46] and incorporate distinct chalcogen atoms on each side.

JTMDs have been successfully synthesized via a modified chemical vapor deposition method [43,45,48], controlled sulfurization [46], and plasma stripping following thermal annealing [46]. Their asymmetric atomic structure offers unique functionalities due to the intrinsic electric field and grants them novel properties like Rashba spin–orbit coupling and piezoelectricity, making them ideal for potential applications in electronics, piezoelectrics, sensors and actuators, and beyond [44,45].

Besides electronic properties, tuning the chemical constitution of the (J)TMD nanomaterials and their hybrid heterostructures has a profound effect on the vibrational properties and other phonon-related phenomena [49,50,51,52,53,54,55,56,57]. Since the symmetry is broken in these structures, the thermal relaxation cannot be described sufficiently by the Peierls–Boltzmann transport equation [58,59]. Due to their relatively large electron band gaps in TMD-based materials, the thermal conductivity is primarily governed by phonon transport.

As previously demonstrated by molecular dynamics simulations of pristine TMDs, an inverse correlation exists between the thermal conductivity (TC) [52] and the width of the phonon band gap (PBG) [50]. In detail, the TC values of few-layer MoS_2_, MoSe_2_, WSe_2_, and WS_2_ have been estimated to be 18.0, 15.5, 14.2, and 11.6 W/mK, and the corresponding phonon band gaps are 0.5, 1.0, 0.9, and 2.4 THz^−1^; see Figure 3 in reference [52]. The thermal conductivity of TMDs such as MoS_2_ and MoSe_2_ is the same along the armchair and zigzag directions [60]. In addition, the TC has been shown [61] to decrease significantly along the normal direction in multilayer TMDs; a similar response is expected for JTMDs as well.

Introducing defects has been shown to affect the phonon spectra upon suppressing existing phonon frequencies and forming new ones [50,62,63]. Localized vacancies in 2D TMDs, created by removing metal or chalcogen atoms, suppress phonon propagation and induce localized phonon states, significantly reducing thermal conductivity [62,63,64]. On the contrary, the formation of substitutional defects affects thermal conductivity depending on a complex interplay between concentration, spatial distribution (e.g., ordered patterns [52] and sharpness of the interfaces [55]), and the type of substituted atoms. For example, the phonon spectrum mismatch and the variation in thermal conductivity at periodic heterostructure media have been shown to suppress heat dissipation, resulting in a substantial decrease in TC at high densities of heterostructure patterns of up to an order of magnitude (down to ca. 2.5 W/mK) [52]. On the other hand, the rate of thermal relaxation in triangular TMD heterostructures exhibits a complicated behavior on the resolution (sharpness) of the heterostructure interface [55].

Despite existing efforts to characterize the thermal properties of JTMDs [57,65,66], the thermal behavior of hybrid JTMD heterostructures remains currently unexplored. The present work expands upon previous research on traditional TMDs [50,52,55] and deals with investigating the thermal relaxation in various bilayer JTMD heterostructures with diverse compositions regarding the type of transition metal (Mo or W) and chalcogen atom (S or Se). Both pristine and hybrid JTMD lateral heterostructures are considered here, the latter featuring triangular regions with different chemical constitution than the surrounding bulk region.

**Figure 1:**
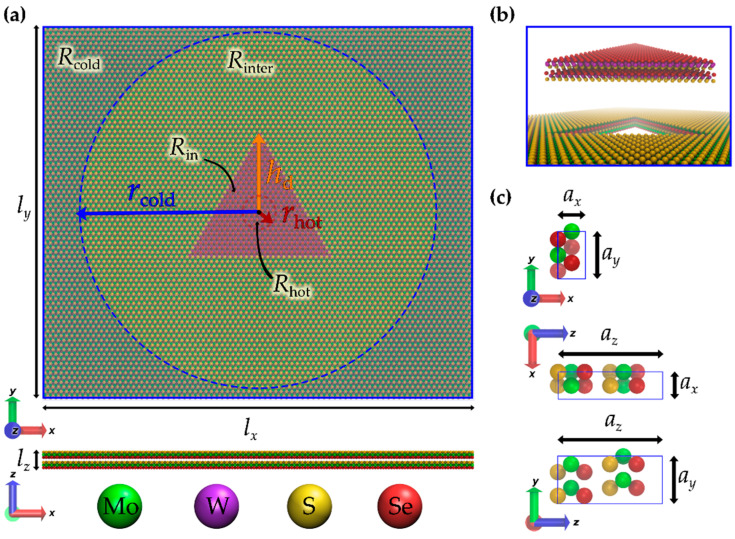
(**a**) Atomistic illustration of a bilayer JTMD heterostructure with (*n_x_*, *n_y_*) = (80, 40) replications of the orthogonal unit cell along the *x*- and *y*-axes, respectively. A top view (**top**) and a side view (**bottom**) are shown, where atoms of different species are depicted by spheres of a particular color. The chemical constitution within the inner triangular region (*R*_in_) with vertex-to-centroid distance *h*_d_ = 5 nm differs from the composition outside of this region (*R*_out_ = *R*_inter_ + *R*_cold_). The temperature is initially fixed to *T*_hot_ in a circular region *R*_hot_ with radius *r*_hot_. In the region *R*_cold_, outside a circle with radius *r*_cold_, the temperature is permanently fixed to *T*_cold_. (**b**) Schematic representations of the atoms within regions *R*_in_ (**top**) and *R*_out_ (**bottom**). (**c**) Illustrations of the orthogonal 12-atom unit cell in the P63 mmc (2H) space group along the −*z*, *y*, and *x* directions.

The outer part of the investigated bilayer nanosheets is kept at an ambient temperature (300 K), while the central part is thermally excited at an elevated temperature, as shown in Figure 1a. Upon removing the heat source at the central part of the material, the local temperature steadily approaches room temperature. By tracking the rate of temperature decrease, we quantify the characteristic relaxation times by fitting with the Kohlrausch–Williams–Watts stretched exponential function [67]. Similar to the cases of low-dimensional nonlinear systems [68,69], laser-excited graphene [70], and various glassy materials [71,72], we find nonexponential relaxation phenomena.

Given the substantial interest in Janus TMDs, this research addresses a critical knowledge gap in their thermal relaxation properties. Notably, we quantify similarities and differences in thermal relaxation between traditional and Janus TMDs, highlighting the influence of inhomogeneous interfaces in these triangular heterostructures. Pristine JTMDs with S and Se outer and Mo middle layers (SMoSe) feature a similar response toward equilibrium to conventional transition metal dichalcogenides. On the contrary, SWSe JTMDs exhibit characteristic relaxation times about twice as large. Engineering lateral heterostructures of JTMDs delays heat dissipation considerably due to phonon spectrum mismatch between the different materials and to the variation in thermal conductivity at the interfaces [50,52,73,74], as was discussed previously. The characteristic relaxation times are of the order of tens or even hundreds of picoseconds for the heterostructures examined here depending on their chemical constitution.

## 2. Materials and Methods

Molecular dynamics simulations were performed with the open-source-code LAMMPS (Large-scale Atomic/Molecular Massively Parallel Simulator). The equations of motion are integrated numerically by employing the velocity-Verlet algorithm [75] with a time step of 1 femtosecond (fs). Interatomic interactions are modeled using harmonic bonds and bond-bending angles, Lennard-Jones dispersive interactions, and Coulomb electrostatic potentials using the parameters from reference [61] (set 9 parameters in Table 3 therein). Long-range interactions are calculated analytically below a cutoff distance of *r*_c_ = 1 nm. Beyond this distance, they are estimated with Ewald’s summation method [76] using the particle–particle/particle–mesh (PPPM) solver [77] in LAMMPS [78]. The relative error in forces was 10^−5^. According to the results presented in Ref. [50], the vibrational spectra of MoS_2_, MoSe_2_, WS_2_, and WSe_2_ using this force field [61] conform with density functional theory (DFT) calculations and are dominated by the corresponding atomic masses.

Figure 1a depicts an atomistic illustration of a JTMD bilayer heterostructure featuring a triangular region *R*_in_ with a vertex-to-centroid distance *h*_d_ = 5 nm having a different chemical composition from the surrounding region; see Figure 1a,b. The structure was generated by replicating the orthogonal 12-atom unit cell (Figure 1c) *n_x_* = 80 and *n_y_* = 40 times along the *x*-axis and *y*-axis, respectively. Notably, the individual atomic layers within the Janus-like monolayers incorporate different chalcogens at the top and bottom atomic layers.

The aim of the numerical simulations performed here is to investigate the thermal relaxation of irradiated JTMDs. To this end, we track the evolution of the local temperature at the central, initially heated region as it tends toward its equilibrium value after the removal of the radiation source.

The simulation begins by assigning initial velocities to all atoms within the simulation box. The velocities of the atoms in the *R*_in_ and *R*_out_ = *R*_inter_ + *R*_cold_ regions were sampled from Boltzmann distributions corresponding to the temperatures *T*_hot_ and *T*_cold_, respectively. Following this, the system underwent equilibration upon maintaining temperature control in a spatially dependent manner. The temperature of the region *R*_hot_ (inside the red circle in Figure 1a) was maintained at *T*_hot_ = 370 K. The temperature of the region *R*_cold_ (outside the blue circle Figure 1a) was maintained at an ambient temperature (*T*_cold_ = 300 K). The intermediate region (*R*_inter_), as well as the remaining part of *R*_in_ apart from its central part *R*_hot_, was not thermostated, allowing for natural heat transfer from *R*_hot_ to *R*_cold_.

In particular, the specimens were simulated for extended time intervals (at least 1 ns) in a hybrid statistical ensemble with a constant number of atoms (*N*), atmospheric lateral pressure (*P* = 1 atm), and partial temperature control. At the beginning of this phase, the system underwent equilibration for 0.1 nanoseconds (ns) using the velocity rescale algorithm. Then, temperatures/pressures were controlled using the Nosé–Hoover thermostat/barostat, respectively [79,80,81]; the corresponding relaxation times were 0.1 ps/1.0 ps.

After reaching a steady state, where a constant energy flux was established, the configurations underwent additional simulation under the same ensemble. Every 300,000 steps (0.3 ns), a snapshot of the system’s trajectory was captured. These snapshots represent different randomized configurations corresponding to the same steady state and were used as starting points for the temperature relaxation experiments. At least 20 such different realizations were considered in our simulations. In particular, for each one of these snapshots *i* (corresponding to the *i*th realization), the thermostating at the central region was removed, resulting in a decline of the local temperature *T_i_*(*t*) of region *R*_hot_ over time toward the equilibrium value *T*_cold_.

The time-dependent local temperature was averaged over all different realizations:

(1) $$\langle T\left(t\right)\rangle =\frac{1}{i_{\max}}\sum_{i=1,i_{\max}}T_{i}\left(t\right)$$

with *T_i_*(*t*) being the evolution of the temperature of the inner region from the *i*th realization and *i*_max_ representing the number of realizations. The thermal relaxation was quantified by fitting a reduced temperature to a Kohlrausch–Williams–Watts stretched exponential function [67]:

(2) $$\tilde{T}\left(t\right)=\exp\left(-{\left[t/t_{K}\right]}^{\beta }\right)$$

where the two fitting parameters *t*_K_ and *β* correspond to the characteristic time scale and the stretched exponent, respectively, while

(3) $$\tilde{T}\left(t\right)=\frac{\langle T\left(t\right)\rangle -T_{\mathrm{cold}}}{T_{\mathrm{hot}}-T_{\mathrm{cold}}}$$

represents the reduced temperature. The mean relaxation time is obtained analytically through direct integration of Equation (2):

(4) $$\tau_{\mathrm{av}}=\int_{0}^{\infty }\tilde{T}\left(t\right)dt=t_{K}\frac{\Gamma \left(1/\beta \right)}{\beta }$$

where Γ is the gamma function. The uncertainty of *τ*_av_ was determined based on the standard deviation of the fitting parameters *t*_K_ and *β* from the diagonal of the covariance matrix [82] resulting upon fitting Equation (2) and applying the error propagation theory:

(5) $$\delta \tau_{\mathrm{av}}=\sqrt{{\left(-\frac{\Gamma \left(1/\beta \right)}{\beta^{2}}t_{K}\delta \beta -\Psi \left(1/\beta \right)\Gamma \left(1/\beta \right)\frac{1}{\beta^{3}}t_{K}\delta \beta \right)}^{2}+{\left(\frac{\Gamma \left(1/\beta \right)}{\beta }\delta t_{K}\right)}^{2}}$$

where $\Psi =\frac{d}{dx}\ln\left(\Gamma \left(x\right)\right)$ is the digamma function [55].

The visualizations of the atomistic configurations were realized with the open-source programs VMD Molecular Graphics Viewer v1.9.4 [83] and the Advanced Image Editor Photopea [84]. The plots were derived with Veusz v3.6.2 [85].

## 3. Results and Discussion

### 3.1. Pristine and Janus Spatially Homogeneous TMDs

In this subsection, the thermal relaxation of spatially homogeneous pristine and Janus TMD materials is presented. Spatial homogeneity is established across the *xy* plane of the 2D nanostructures. The material composition of these TMDs plays a crucial role in dissipating thermal energy and the corresponding thermal relaxation.

**Figure 2:**
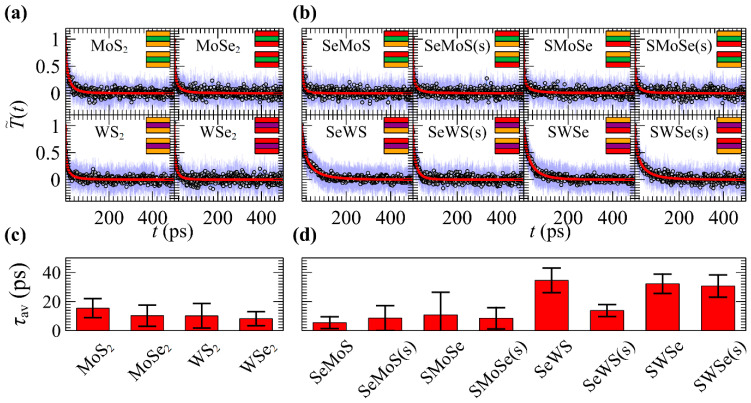
Evolution of the average reduced temperature (circles) through Equations (1) and (3) during the course of the relaxation simulations of (**a**) pristine TMDs and (**b**) JTMDs; blue error bars correspond to the standard deviation. Red lines indicate fittings with Equation (2). The presence of trailing “(s)” in the notation indicates mirror symmetry of the individual sheets that comprise the bilayer along the *xy* plane. The insets depict side-view schematics of the corresponding bilayer structures following the color code: Mo (green), W (purple), S (orange), and Se (red). (**c**,**d**) The mean relaxation times (*τ*_av_) as obtained from the fitting of the reduced temperature shown in (**a**,**b**), respectively, with a stretched exponential function (Equation (2)). Error bars depict the standard deviations from Equation (5).

Figure 2a depicts the variation of reduced temperature (calculated through Equations (1) and (3)) during the relaxation toward the equilibrium of pristine TMD materials. Figure 2b presents the corresponding variations for JTMDs using the notation *XMY* and *XMY*(s). Here, *X* and *Y* denote the chalcogen atoms at the top and bottom atomic layers of each sheet, while *M* represents the metal atom at the middle atomic layer. The trailing (s) in the notation indicates the relative stacking order in the bilayer; in particular, it denotes whether the individual sheets forming the bilayer exhibit mirror symmetry across their midpoint along the *z*-axis.

The mean relaxation times *τ*_av_ corresponding to the cases presented in (a) and (b) are shown in Figure 2c,d. *τ*_av_ is calculated through the second equality of Equation (4), where the parameters *t*_K_ and *β* are obtained by fitting the average reduced temperature with the stretched exponential of Equation (2). Error bars represent the standard deviation resulting from Equation (5).

According to Figure 2c, pristine TMDs exhibit very fast characteristic relaxation times, around 8–16 picoseconds (ps), in accordance with the findings of reference [55]. Interestingly, JTMDs containing Mo metal atoms and S/Se chalcogens exhibit a similar response to pristine TMDs, yielding relaxation times within the same range and uncertainty, e.g., compare the relaxation times of pristine MoS_2_ and MoSe_2_ in Figure 2c with the JTMDs relaxation times of the various combinations of Mo with Se/S (first four bars in Figure 2d). On the contrary, JTMDs with W metal atoms display significantly slower relaxation. For instance, heat relaxation is most delayed, (ca. 15–30 ps) in the SeWS and SWSe bilayers (Figure 2d), characterized by the Se and S elements on the outer surface and the W element on the middle layer. Note that the SeWS and SWSe configurations, as well as the SeMoS and SMoSe ones, are equivalent due to their layer arrangement (e.g., compare the first and third columns in the insets of Figure 2b), feature the same relaxation times within the uncertainty of the error bars. On the contrary, the relative orientation of the monolayers appears to affect the relaxation time in a single case, e.g., compare the SeWS and SeWs(s) structures.

### 3.2. JTMDs Lateral Heterostructures

**Figure 3:**
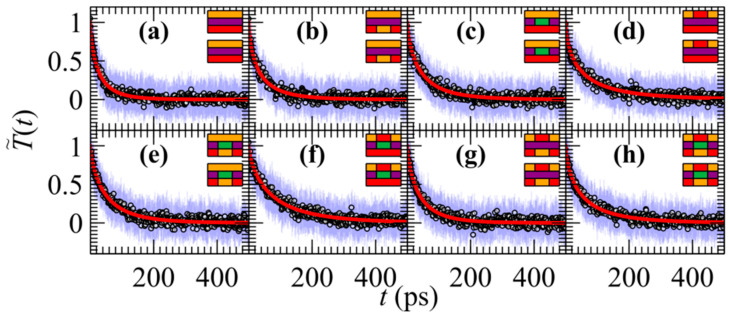
Evolution of the average reduced temperature (circles) through Equations (1) and (3) during the relaxation of bilayer JTMDs lateral heterostructures with pristine and Janus triangular regions; blue error bars correspond to the standard deviations. Red lines indicate fittings with Equation (2). The outer region (*R*_out_ = *R*_inter_ + *R*_cold_) is composed of SWSe in all cases. The composition of the first, second, and third atomic layers in the inner region of the heterostructure (*R*_in_) in panels (**a**–**h**) is illustrated by the side-view schematics of the bilayers in the insets following the color code: Mo (green), W (purple), S (orange), and Se (red).

**Figure 4:**
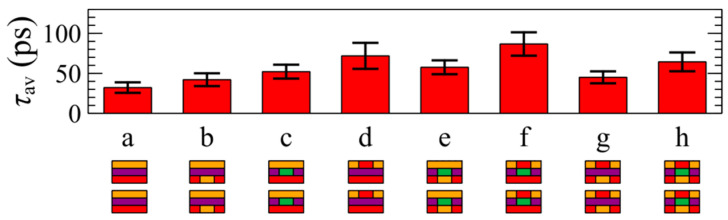
The mean relaxation times for cases (**a**–**h**) shown in Figure 3. Error bars depict the standard deviations from Equation (5). The composition of the first, second, and third atomic layers in the inner region of the heterostructure (*R*_in_) is illustrated by the side-view schematics of the bilayers in the insets following the color code: Mo (green), W (purple), S (orange), and Se (red).

Here, the thermal relaxation of lateral JTMD heterostructures is considered. These materials are no longer spatially homogeneous across the *xy* plane of the 2D nanostructure. In Figure 3, the time dependence of the average reduced temperature during the numerical simulation of JTMD heterostructures is depicted. The outer region (*R*_out_ *= R*_inter_ + *R*_cold_; see Figure 1a) comprises Janus SWSe in these structures. The composition of the three distinct atomic layers (the first corresponds to the upper layer, second to the middle, and third to the lower layer) in the inner region (*R*_in_) of the heterostructure is represented by the side-view schematics of the bilayer in the insets of Figure 3, with Mo (green), W (purple), S (orange), and Se (red) color codes. Figure 4 presents the mean relaxation times *τ*_av_ corresponding to the cases shown in Figure 3, with the error bars representing the standard deviations from Equation (5).

It is noted that any substitution on the upper, middle, or lower layer results in an increase in the mean relaxation time. For instance, the spatially homogeneous material without any substitution (label a in Figure 4) exhibits a relaxation time of 30–40 ps, while the next shown heterostructure (label b) has a relaxation time slightly higher (ca. 50 ps). Furthermore, the relaxation time of the heterostructure labeled f is nearly 2.5 times longer than that of the structure without any substitutions. However, performing more substitutions does not necessarily result in longer mean relaxation times. For example, the heterostructure h does not exhibit longer *τ*_av_, nor does the g material feature longer relaxation times than the d lateral heterostructure.

**Figure 5:**
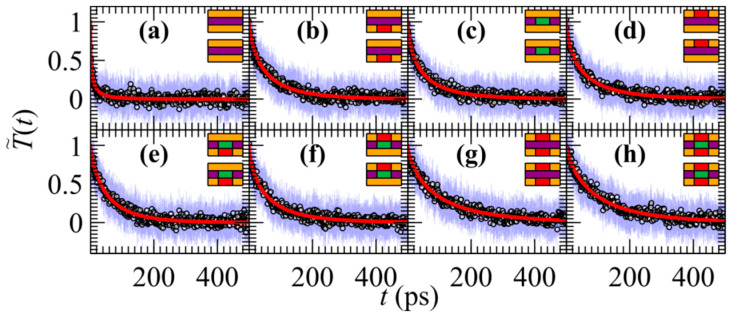
Evolution of the average reduced temperature (circles) through Equations (1) and (3) during the relaxation of bilayer JTMD lateral heterostructures with pristine and Janus triangular regions; blue error bars correspond to the standard deviations. Red lines indicate fittings with Equation (2). The outer region (*R*_out_ = *R*_inter_ + *R*_cold_) is composed of WS_2_ in all cases. The composition of the first, second, and third atomic layers in the inner region of the heterostructure (*R*_in_) in panels (**a**–**h**) is illustrated by the side-view schematics of the bilayers in the insets following the color code: Mo (green), W (purple), S (orange), and Se (red).

**Figure 6:**
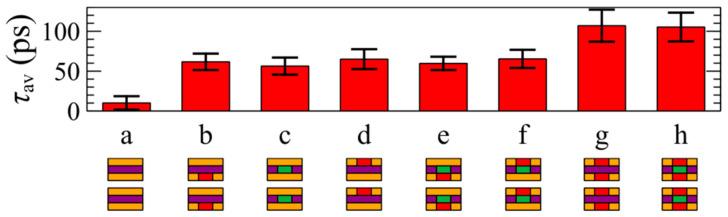
The mean relaxation times for cases (**a**–**h**) shown in Figure 5. Error bars depict the standard deviations from Equation (5). The composition of the first, second, and third atomic layers in the inner region of the heterostructure (*R*_in_) is illustrated by the side-view schematics of the bilayers in the insets following the color code: Mo (green), W (purple), S (orange), and Se (red).

Finally, JTMD lateral heterostructures obtained by similar substitutions in pristine transition metal dichalcogenides are considered. The obtained thermal relaxation results are shown in Figure 5. The outer region (*R*_out_ *= R*_inter_ + *R*_cold_) consists of pristine WS_2_ now. The composition of the first, second, and third layers in the inner region (*R*_in_) is denoted by the insets based on the same color code as explained above. Figure 6 presents the mean relaxation times *τ*_av_ for the dynamics shown in Figure 5, with error bars indicating the standard deviations. Again, it is evident that altering atoms in any of the three atomic layers in the inner region of the heterostructure leads to a significant increase in relaxation time. As an example, the relaxation time of the pristine WS_2_ (label a in Figure 6) is approximately 10 ps, whereas for the heterostructure labeled c (indicating the presence of Mo atoms in the middle layer of *R*_in_), the relaxation time is around 50 ps, which is five times longer. Furthermore, the lateral heterostructures within this category labeled as g or h demonstrate the longest relaxation times, about one order of magnitude longer than that of the pristine structure.

As our results demonstrate, the relaxation of thermal excitations is substantially influenced by the material composition of the JTMD nanostructures. Lateral heterostructures of Janus transition metal dichalcogenides can increase the thermal relaxation times by an order of magnitude as compared to that of spatially homogeneous pristine TMDs. Altering atoms in more than one layer does not necessarily imply longer relaxation times than in structures with fewer layer substitutions. In general, the values of thermal relaxation times in JTMD lateral heterostructures fall in the same regime as the corresponding ones in conventional TMD heterostructures [55].

## 4. Conclusions

This study investigates the in-plane thermal relaxation of various Janus transition metal dichalcogenide bilayers with triangular heterostructures using molecular dynamics simulations. Our numerical simulations explore the effect of composition (transition-metal and chalcogen atoms) on thermal behavior in both pristine JTMD and hybrid JTMD lateral heterostructures.

The samples were subjected to non-equilibrium molecular dynamics simulations within a hybrid statistical ensemble. This ensemble allows for spatially dependent temperature control, resulting in a temperature gradient across the material. A central circular region was designated as the heat source and was maintained at a higher temperature. Conversely, the outer region of the material acted as a heat sink and was held at a cooler (room) temperature. The intermediate region remained uncoupled from the thermostats, allowing for natural heat transfer from the “hot” center toward the “cooler” edges. Once a steady-state condition was achieved (characterized by a constant energy flux across the material), the thermostat controlling the central region was deactivated. This sudden removal of the heat source resulted in a gradual decrease in the local temperature at the central hot region of the JTMD bilayer. The characteristic relaxation time was determined upon fitting the time dependence of the local temperature at the center of the structure with the Kohlrausch–Williams–Watts stretched exponential function.

The findings demonstrate that engineering lateral heterostructures in JTMDs significantly delays heat dissipation compared to pristine JTMDs. This delay is attributed to the mismatch in vibrational frequencies and variations in thermal conductivity between the different materials within the heterostructure. The characteristic relaxation times for the examined heterostructures can be enhanced by up to an order of magnitude, depending on their specific chemical composition. The thermal properties of bilayer TMDs closely resemble those of their few-layer counterparts while monolayer TMDs exhibit distinct, enhanced relaxation behavior [55]; we anticipate an analogous response for JTMDs as well.

These results highlight the potential for manipulating thermal properties in JTMDs through structural engineering, paving the way for advanced applications in heat management, nanophononics, thermal devices, and optoelectronics. For instance, forming JTMD heterostructures with low thermal conductivity can enhance the efficiency of potential thermoelectric devices. This is because the figure of merit (ZT) improves when thermal conductivity decreases while maintaining electrical conductivity. Synthesizing JTMDs with tailor-made thermo-mechanical properties offers potential for applications in nuclear reactor fuel materials by enabling controlled heat distribution during the fuel cycle [86]. Controlling phonon transport along the JTMD surface holds promise for developing advanced interconnect components (resonators, waveguides, and switches) in quantum computing applications and next-gen phononic transistors [62,87].

## Data Availability

The raw data supporting the conclusions of this article will be made available by the authors upon request.
